# A clinical dataset on type-2 diabetes including demographic, anthropometric, and biochemical parameters from Bangladesh

**DOI:** 10.1016/j.dib.2026.112457

**Published:** 2026-01-10

**Authors:** Md. Younus Bhuiyan, Shahriar Siddique Ayon, Md. Ebrahim Hossain, Md. Saef Ullah Miah, Afjal H. Sarower, Fateha khanam Bappee

**Affiliations:** a Department of Computer Science, Daffodil International University, 1207 Dhaka, Bangladesh; b Department of Computer Science, American International University-Bangladesh (AIUB), 1229 Dhaka, Bangladesh; c Department of Computer Science and Telecommunications Engineering (CSTE), Noakhali Science and Technology University (NSTU), 3814 Noakhali, Bangladesh

**Keywords:** Type-2 diabetes, Clinical dataset, Biomedical data, Bangladesh, Healthcare analytics

## Abstract

Type-2 diabetes is a major public health concern in Bangladesh, and this dataset provides 1065 curated patient records with demographic, anthropometric, and clinical variables relevant to its assessment. The data were collected during routine clinical visits and recorded by trained staff, with checks to ensure accuracy and completeness. It includes basic details like age, pregnancy count, body mass index, and skin-fold thickness; vital signs such as blood pressure; lab results related to blood sugar (fasting glucose and insulin); the Diabetes Pedigree Function; and a simple yes/no label for Type-2 diabetes. A few values are missing for diastolic blood pressure and skin-fold thickness, so users should handle these carefully. Since the data are cross-sectional and come from patients seeking care, there are more diabetic cases (840) than non-diabetic cases (225). The dataset is intended for reuse in method development (for example, machine-learning classifier training, feature-selection benchmarking, and oversampling/imputation research), for context-specific epidemiologic description and model validation in South Asian clinical settings, and as a teaching resource for reproducible biomedical-data workflows.

Specifications Table

**Table:** 

Subject	Health Sciences, Medical Sciences & Pharmacology
Specific subject area	Diabetes research, clinical epidemiology, biomedical informatics
Type of data	Raw, Processed,Tabular (CSV)
Data collection	Collected from 1065 patients at Narsingdi Diabetic & General Hospital, Bangladesh, using hospital records, clinical measurements, and structured patient interviews. Includes 840 diabetic and 225 non-diabetic patients, with data on serum insulin (324 with, 741 without) and genetic predisposition (689 with, 376 without). Stored as a de-identified CSV file.
Data source location	Narsingdi Diabetic & General Hospital, Narsingdi, Bangladesh (Latitude: 23°55′37″ N, Longitude: 90°43′9″ E)
Data accessibility	**Repository name:** Mendeley Data **Data identification number (DOI):**10.17632/rn9m3zb7nt.1 **Direct URL to data:**https://data.mendeley.com/datasets/rn9m3zb7nt/1 **Instructions for accessing these data:** The dataset is licensed under CC BY 4.0 and is freely available for download without registration. Includes one UTF-8 encoded CSV file named
Related research article	*None*

## 1 Value of the Data

• The dataset comprises 1065 clinically validated patient records with key demographic, anthropometric, and biochemical variables, offering a comprehensive and reliable foundation for Type-2 diabetes analysis and risk modeling.
• The dataset reflects region-specific diabetes patterns in Bangladesh, addressing a key gap in publicly available South Asian data.
• The class imbalance and minimal missing data make this dataset well suited for testing machine learning methods such as feature selection, oversampling, imputation, and model robustness analysis.
• The dataset is a clean, well-documented CSV with clearly defined variables and units, enabling reproducible research and benchmarking of diabetes prediction models.
• The dataset also supports biomedical data science education, evidence-based healthcare planning, and local screening and resource allocation in low- and middle-income setting

## 2 Background

Type 2 diabetes is today regarded as one of the chronic diseases with the fastest rate of growth in the globe, and it has become a significant global health concern. International health organizations report that hundreds of millions of people worldwide today have diabetes, with Type 2 diabetes accounting for 90–95 % of cases and by 2045, that number is expected to increase to over 780 million [1]. Type 2 diabetes can cause severe complications like heart disease, stroke, kidney failure, nerve and vision damage, and foot issues that may lead to amputation [2]. Both low- and middle-income countries are seeing an increase in the prevalence, which has been attributed to factors such as decreased physical activity, poor diets, and increasing urbanization [3]. Despite the difficulty, there are still few publicly accessible and clinically certified datasets from South Asia [4]. The majority of diabetes datasets currently available are based on Western populations, which could not adequately represent the environmental, lifestyle, and genetic risk factors particular to South Asian groups [5,6]. The lack of region-specific datasets, however, limits the creation of precise healthcare plans and efficient predictive models. By gathering clinically validated demographic, anthropometric, and biochemical data from patients in Bangladesh, this dataset was created to close this gap.

## 3 Data Description

This dataset captures clinical, demographic, and biochemical information from patients to study Type 2 diabetes. It includes details such as age, (BMI), blood pressure, insulin and glucose levels, skin thickness, pregnancy history, and genetic risk factors, along with each patient’s diabetes status. Table 1 provides a detailed overview of the variables, including their names, descriptions, data types, and corresponding units or categories.

**Table 1:** Summary of variables in the type 2 diabetes patient dataset.

Variable Name	Description	Data Type	Units / Categories
No. of Pregnancy	Number of times the patient has been pregnant	Integer	Count (0, 1, 2, …)
Age	Age of the patient	Integer / Float	Years
BMI	Body Mass Index of the patient	Float	kg/m²
BP(Systolic)	Systolic blood pressure	Integer / Float	mmHg
BP(Diastolic)	Diastolic blood pressure	Integer / Float	mmHg
DiabetesPedigreeFunction	Genetic risk of diabetes based on family history	Float	Dimensionless (0–2+)
Insulin	Blood insulin level	Float	µU/mL
Skin Thickness(mm)	Triceps skin fold thickness	Float	Millimetres (mm)
Type-2 Diabetic	Diabetes status	Binary / Categorical	0 = Non-diabetic, 1 = Diabetic
Glucose	Plasma glucose concentration	Integer / Float	mg/dL

## 4 Experimental Design, Materials and Methods

### 4.1 Study setting and participants

Between August and October 2024, we carried out a cross-sectional study at Narsingdi Diabetic & General Hospital, a key healthcare centre in Bangladesh that serves both urban and rural communities. During this period, all patients visiting the outpatient clinic were invited to take part in the study. To be eligible, participants had to be over 20 years old, attending the hospital for routine care, and willing to provide informed consent (with consent from parents or guardians for younger patients where necessary). Patients with serious health conditions not related to diabetes were excluded.

We collected information through face-to-face interviews, where trained staff entered responses directly into a structured CSV file on a password-protected laptop. To ensure privacy, we did not record any personally identifiable details. In total, 1065 people took part in the study, including 840 patients with type 2 diabetes and 225 individuals without diabetes who were included as a comparison group. The dataset comprises 1065 rows and 10 columns, is provided in CSV format, and uses UTF-8 encoding, offering a clear and accessible structure for reproducibility and analysis. Once data collection was completed, doctors and hospital authorities carefully reviewed the dataset to check for accuracy, consistency, and clinical validity before final approval.

### 4.2 Data collection procedures

**Fig. 1:**
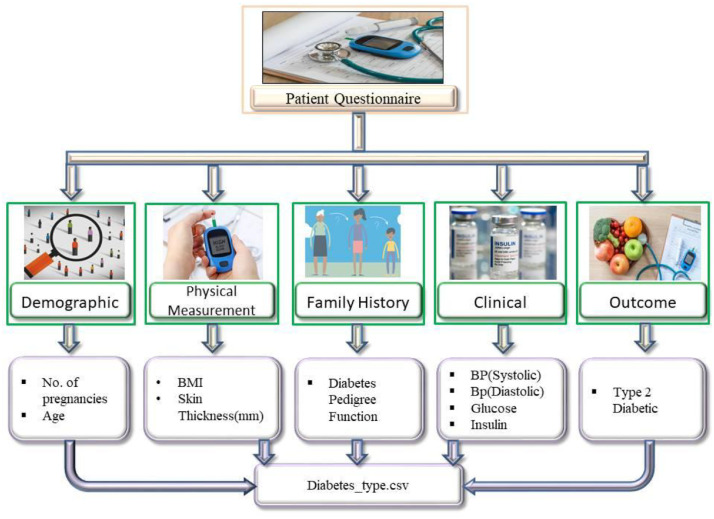
Overview of dataset features and their relationship with Type 2 Diabetes status. The figure shows each feature’s distribution across five categories, summarizing the dataset’s categorical composition.

This dataset includes information from both diabetic and non-diabetic patients, covering 10 key features. For each participant, we collected demographic details (such as age and number of pregnancies), physical measurements (including BMI and skin thickness), and family history of diabetes (Diabetes Pedigree Function). To ensure reproducibility, we provide details of the instruments used in the study. Fasting plasma glucose was measured using the HemoCue Glucose 201+ point-of-care analyzer on venous samples. Serum insulin levels were determined with enzyme-linked immunosorbent assay (ELISA) using Mercodia Insulin ELISA kits in the hospital laboratory. Blood pressure, both systolic and diastolic, was recorded using an automated oscillometric sphygmomanometer following standard clinical guidelines. The presence or absence of type 2 diabetes was confirmed by doctors based on medical reports and diagnoses. Fig. 1, provides an overview of the dataset, highlighting the various features collected from participants—including demographic details, physical measurements, clinical information, and family history—along with their Type 2 diabetes status.

**Fig. 2:**
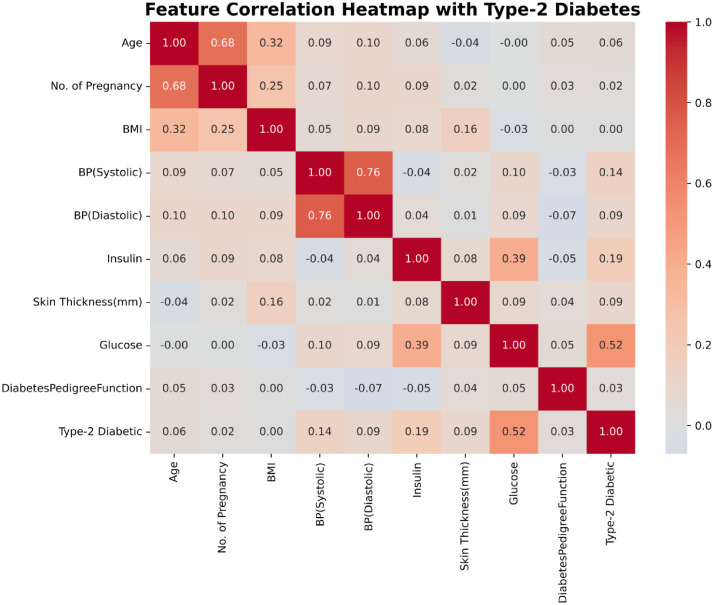
Correlation Heatmap of dataset features. The figure shows pairwise feature correlations in the Type 2 Diabetes dataset for analysis and feature selection.

The final dataset included 1065 participants, of whom 78.87 % had type 2 diabetes and 21.13 % were non-diabetic, with ages ranging from 21 to 86 years. Fig. 2, shows a correlation Heatmap illustrating how different features in the Type 2 Diabetes dataset relate to each other. Darker red shades indicate stronger positive relationships, while lighter colors show weaker or negative correlations. The diagonal values (1.00) represent each feature’s perfect correlation with itself. Age shows a strong correlation with the number of pregnancies (0.68), reflecting expected demographic patterns. Systolic and diastolic blood pressures are also closely related (0.76), as anticipated due to their physiological link. Among the clinical measures, glucose stands out with the strongest positive correlation with type 2 diabetes (0.52), followed by insulin (0.19) and systolic blood pressure (0.14), while BMI and skin thickness exhibit very weak associations. Interestingly, age, number of pregnancies, and the diabetes pedigree function show almost no correlation with diabetes in this dataset, suggesting that biochemical markers particularly glucose are the most influential predictors.

**Fig. 3:**
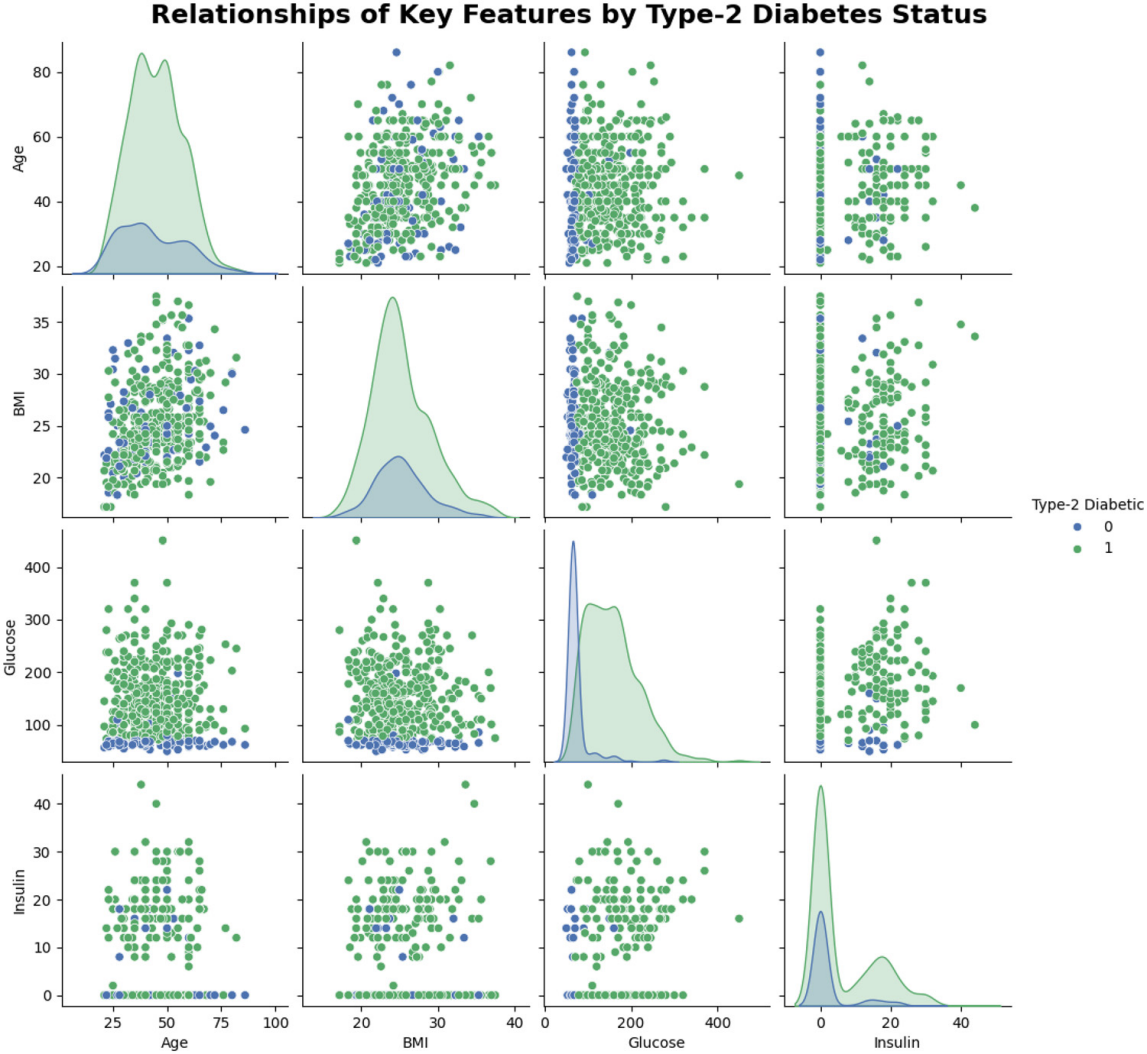
Relationship between key features (Age, BMI, Glucose, Insulin) and Type 2 Diabetes status. Points are colored by diabetes status (blue for non-diabetic, green for diabetic) to show feature distribution across classes.

Fig. 3, shows a pair-plot that explores the relationships among key features such as Age, BMI, Glucose, and Insulin in the Type 2 Diabetes dataset. Each point represents an individual, with blue indicating non-diabetic (0) and green indicating diabetic (1). The diagonal plots display the distribution of each feature, while the scatter plots reveal how pairs of features interact, highlighting possible patterns or differences between the two groups. Diabetic participants (green) generally exhibit higher glucose levels than non-diabetic participants (blue), highlighting glucose as the most distinguishing feature. Age distributions indicate that diabetes is more common among older individuals, although some younger participants are also affected. BMI shows overlapping distributions between the two groups, with diabetics tending to have slightly higher values, suggesting a modest association. Insulin levels are highly variable, with many participants in both groups near zero, possibly reflecting measurement variability or effects of treatment. Overall, while weak linear trends are observed between BMI, glucose, and insulin, glucose provides the clearest separation between diabetic and non-diabetic participants.

### 4.3 Data preprocessing

The dataset was initially loaded and carefully inspected for completeness and consistency. To ensure data quality, all entries were cross verified by independent reviewers. Participants’ demographic, clinical, and family history information was systematically organized. Some missing values were observed in the dataset: 3 missing entries in the ‘BP (Diastolic)’ feature and 7 missing entries in the ‘Skin Thickness (mm)’ feature. These missing values were not preprocessed and remain as null values in the dataset. The target variable, ‘Type-2 Diabetic’, was encoded to distinguish between diabetic and non-diabetic participants, and the distribution of each class was examined to assess class balance. Continuous variables, including ‘Age’, ‘BMI’, and other clinical measurements, were analyzed to determine their ranges and identify potential outliers. Additionally, diabetes status was encoded in a binary format to facilitate further analysis. After final checks and necessary preprocessing, the cleaned dataset was saved as a CSV file for subsequent analysis and research use.

### 4.4 Previous studies

While several publicly accessible diabetes datasets have been extensively utilized in computational and biological research, the majority come from non-South Asian or Western populations. One well-known example is the PIMA Indians Diabetes Dataset, which was created in the US and includes data on 768 Pima Indian women [7]. The PIMA Indians Diabetes Dataset mainly reflects Western risk profiles and may not capture factors unique to South Asians. Our dataset of 1065 Bangladeshi patients includes additional biochemical and familial features, enabling region-specific analyses and more equitable predictive models. A clinical study at AIIMS Bathinda found that type 2 diabetes affects age groups differently: younger adults (20–40 years) show higher HbA1c levels, middle-aged individuals (41–60 years) have elevated triglycerides, and those over 40 experiences more neuropathy [8]. Type 2 diabetes mellitus (T2DM) poses a growing public health challenge in South Asia, marked by earlier onset at lower BMI, increased abdominal and ectopic fat, and faster β-cell decline—leading to rapid glycemic worsening and heightened risk of complications [9]. A prospective clinical trial in Taiwan involving 35 T2D patients demonstrated that three months of tempeh capsule supplementation effectively lowered HbA1c and triglyceride levels [10]. In another large-scale investigation, outcomes from 5325 T2D patients across 14 randomized, placebo-controlled dapagliflozin studies were combined and assessed using longitudinal mixed-effects models, providing robust evidence for both efficacy and safety of the intervention [11]. Complementing these clinical insights, a curated T2D disease–gene association dataset was developed from PubMed abstracts using text mining and manual validation to support classifier training, offering a valuable resource for computational modeling and biomarker discovery [12].

### 4.5 Highlighting the dataset’s value

Table 2 provides an overview of existing diabetes datasets, summarizing their sample sizes and key limitations. Most prior datasets primarily focus on a limited set of laboratory or demographic values, often lacking biochemical detail or family history information. In contrast, our dataset includes 10 clinically validated variables covering demographic, anthropometric, and biochemical dimensions. The inclusion of features such as insulin levels, skin thickness, blood pressure, and family history (Diabetes Pedigree Function) alongside age, BMI, and glucose measurements provides a richer and more comprehensive representation of type-2 diabetes risk factors. This broader scope enables more robust machine learning applications and supports holistic research into both clinical and lifestyle contributors to diabetes within a South Asian population. This dataset can advance diabetes research in resource-limited settings by enabling region-specific risk prediction using South Asian–specific features, such as lower BMI thresholds and familial pedigree functions. Its demographic diversity and class imbalance also support fairness analyses in AI tools, allowing evaluation of bias mitigation strategies (e.g., oversampling techniques) for equitable screening across different populations in Bangladesh. Overall, this dataset provides a comprehensive, region-specific resource for diabetes research, model development, fairness testing, and educational or clinical applications.

**Table 2:** Comparison of diabetes datasets used in existing studies.

No.	Reference	Repository	Number of Samples	Limitation of Dataset
1	[7]	Hospital Collected Data	5288 patients	The dataset is limited by a relatively low diabetes prevalence of 6.5 % (342 of 5288 participants).
2	[8]	Self-report Questionnaires	100 patients	The study was limited to 100 randomly selected type 2 diabetes patients aged 40–65.
3	[10]	Kaohsiung Veterans General HospitalQuestionnaires	44 patients	35 eligible participants were enrolled in the study.
4	[11]	AstraZeneca's website	5325 samples	The data contribute to understand the effects of dapagliflozin treatment in patients with type 2 diabetes with and without anemia.
5	[12]	Mendeley Data	142,529 rows	Only Four columns (“PMID”, “Title”, “Date”, and “Abstract text”)
6	Our Dataset [13]	Mendeley Data	1065 samples	Collected from only one tertiary hospital in Narsingdi, Bangladesh, which limits the generalizability of the findings to other regions or healthcare settings

## Limitations

Our dataset was collected from a single tertiary hospital in Narsingdi, Bangladesh, which means the findings may not fully reflect the wider population or other healthcare settings across the country. Some important laboratory tests, such as HbA1c and lipid profiles, were not included because of limited resources. This makes it difficult to assess long-term blood sugar control or estimate cardiovascular risks. Since the study is cross-sectional, it only provides a snapshot in time and cannot show changes over time or establish cause-and-effect relationships. The sample also has more diabetic patients compared to non-diabetic patients, which could influence the accuracy of predictive models unless carefully adjusted. Additionally, a few measurements—such as diastolic blood pressure and skin-fold thickness—had missing or variable data, and while these were curated, they may still introduce some uncertainty in the analyses.

## Ethics Statement

The research protocol received approval from the medical and administrative authorities of Narsingdi Diabetic & General Hospital, Narsingdi, Bangladesh. All procedures involving human subjects adhered to the ethical guidelines set by institutional and national research committees, as well as the principles outlined in the 1964 Helsinki Declaration and its subsequent revisions. Participation was entirely voluntary, with no collection of personally identifiable information. To protect participant confidentiality, all data were anonymized at the time of collection. Informed consent was obtained from each participant; consent was secured from a parent or legal guardian. The dataset was developed solely for academic and research use and contains no sensitive or clinical information that could identify individual patients.

## Credit Author Statement

**Md. Younus Bhuiyan:** Investigation, Methodology, Writing; **Shahriar Siddique Ayon:** Conceptualization, Methodology, Original draft preparation; **Md. Ebrahim Hossain:** Data curation, Writing, Investigation; **Md. Saef Ullah Miah:** Supervision, Writing- Reviewing and Editing; **Afjal H. Sarower:** Software, Visualization; **Fateha khanam Bappee:** Methodology, Writing.

## Data Availability

Mendeley DataType-2 Diabetes Dataset Bangladesh (Original data).
